# Supplementary material for: Web-Based Reporting of Post-Vaccination Symptoms for Inactivated COVID-19 Vaccines in Jordan: A Cross-Sectional Study
Source: Vaccines (Basel). 2022 Dec 25;11(1):44. doi: 10.3390/vaccines11010044 (PMC9866303; doi:10.3390/vaccines11010044)
Supplement: Supplementary file 1 [file vaccines-11-00044-s001.zip › vaccines-2046597-supplementary.pdf]

Short-term side effects of the inactivated COVID-19 vaccine in Jordan

Informed Consent Form:

Dear participant,
This cross-sectional survey aims to collect data on the short-term side effects following the inactivated COVID-19 vaccine. The survey contains two sections, which will take approximately five minutes to answer.
No physical or emotional risks are expected, nor is there any risks resulting from participation in the study.
The data will be used by the researchers exclusively for the purposes of research. The data will not be made available or used by other institutions or used for other purposes. The obtained data will be processed and analyzed for the duration of the project. Upon completion of the project, the data obtained will be encrypted and securely stored as part of measures to ensure the integrity of the research.
Your participation in this research study is voluntary. You may choose not to participate. If you decide to participate in this research survey, you may withdraw at any time.

Thanks in advance for your help and cooperation.
Your participation in completing this questionnaire is greatly appreciated.

Electronic Consent:

Please select your choice below.

Clicking on the "agree" button below indicates that:

1. You have read the above information.
2. You voluntarily agree to participate.

If you do not wish to participate in the research study, please decline participation by clicking on the "disagree" button.

- ❑ Agree
- ❑ Disagree

Section One: Demographic data	
Gender:	Male<br>Female
Age:	18-29 years old<br>30-39 years old<br>40-49 years old<br>50-59 years old<br>≥ 60 years
Marital Status:	Married<br>Single<br>Widowed<br>Divorced
The governorate you live in:	Amman (the capital)<br>Irbid<br>Zarqa<br>Balqa<br>Mafraq<br>Karak<br>Madaba<br>Jerash<br>Ajloun<br>Aqaba<br>Ma'an<br>Tafilah
Nationality:	Jordanian<br>Non-Jordanian
Educational level:	Primary school<br>High school<br>Diploma<br>Bachelor's degree<br>Postgraduate degree (Master's or PhD)
Employment:	Employed<br>Not employed<br>Retired
Are you a smoker?	Yes<br>No<br>Previous smoker

**<u>Section Two: COVID-19 experience</u>**	
Have you been infected with COVID-19 before receiving the vaccine?	Yes<br>No<br>Not sure
In which month did you receive the COVID-19 vaccine?	January 2021<br>February 2021<br>March 2021<br>April 2021<br>May 2021<br>June 2021<br>July 2021<br>August 2021
Did you experience any side effects following the second dose of the COVID-19 vaccine?	Yes<br>No
Select the side effects that you experienced after the first dose[*,#]	- Sore arm at the site of injection<br>- Injection site swelling<br>- Redness at the injection site<br>- Discomfort feeling<br>- Fatigue<br>- Flu-like symptoms<br>- High temperature<br>- Chills<br>- Headache<br>- Shortness of breath<br>- Cough<br>- Muscles/joint pain<br>- Gastrointestinal symptoms such as nausea, vomiting, and diarrhea<br>- Sore throat<br>- Eye pain<br>- Runny nose<br>- Loss or change in the sense of taste or smell<br>- Anorexia<br>- Chest pressure/pain<br>- Drowsiness<br>- Hair loss<br>- Tachycardia or heart palpitations<br>- Lymphadenopathy
Did you receive the second dose of the inactivated COVID-19 vaccine?	Yes<br>No
Did you experience any side effects following the second dose of the COVID-19 vaccine?	Yes<br>No
Select the side effects that you experienced after the second dose[*,#]	- Sore arm at the site of injection<br>- Injection site swelling<br>- Redness at the injection site<br>- Discomfort feeling<br>- Fatigue

	- Flu-like symptoms<br>- High temperature<br>- Chills<br>- Headache<br>- Shortness of breath<br>- Cough<br>- Muscles/joint pain<br>- Gastrointestinal symptoms such as nausea, vomiting, and diarrhea<br>- Sore throat<br>- Eye pain<br>- Runny nose<br>- Loss or change in the sense of taste or smell<br>- Anorexia<br>- Chest pressure/pain<br>- Drowsiness<br>- Hair loss<br>- Tachycardia or heart palpitations<br>- Lymphadenopathy
Did you experience any severe side effects that required hospital admission within four weeks of receiving the inactivated COVID-19 vaccine?	Yes<br>No
* Only participants who answered "*Yes*" in the previous question were able to answer this question.<br># Participants were able to select more than one answer.	
